# Searching for Face-Category Representation in the Avian Visual Forebrain

**DOI:** 10.3389/fphys.2019.00140

**Published:** 2019-02-27

**Authors:** William James Clark, Blake Porter, Michael Colombo

**Affiliations:** Department of Psychology, University of Otago, Dunedin, New Zealand

**Keywords:** conspecific recognition, extrastriate cortex, tectofugal pathway, face-selective neurons, face cell, face-category, pigeon visual forebrain, object representation

## Abstract

Visual information is processed hierarchically along a ventral (‘what’) pathway that terminates with categorical representation of biologically relevant visual percepts (such as faces) in the mammalian extrastriate visual cortex. How birds solve face and object representation without a neocortex is a long-standing problem in evolutionary neuroscience, though multiple lines of evidence suggest that these abilities arise from circuitry fundamentally similar to the extrastriate visual cortex. The aim of the present experiment was to determine whether birds also exhibit a categorical representation of the avian face-region in four visual forebrain structures of the tectofugal visual pathway: entopallium (ENTO), mesopallium ventrolaterale (MVL), nidopallium frontolaterale (NFL), and area temporo-parieto-occipitalis (TPO). We performed electrophysiological recordings from the right and left hemispheres of 13 pigeons while they performed a Go/No-Go task that required them to discriminate between two sets of stimuli that included images of pigeon faces. No neurons fired selectively to only faces in either ENTO, NFL, MVL, or TPO. Birds’ predisposition to attend to the local-features of stimuli may influence the perception of faces as a global combination of features, and explain our observed absence of face-selective neurons. The implementation of naturalistic viewing paradigms in conjunction with electrophysiological and fMRI techniques has the potential to promote and uncover the global processing of visual objects to determine whether birds exhibit category-selective patches in the tectofugal visual forebrain.

## Introduction

Birds derived their visual forebrain structures from an archosaur reptile (diapsid amniotes including living crocodilians and extinct dinosaurs) roughly 320 million years ago (Jarvis et al., 2005). Accordingly, the avian visual system exhibits a nuclear organisation that bears almost no resemblance to the six-layered mammalian neocortex, and is instead composed of densely clustered neuronal cell bodies (Reiner et al., 2004; Briscoe et al., 2018). The organisation of the mammalian neocortex is heavily implicated with the emergence of mammals’ advanced capability to integrate complex sensory information for the robust perception and recognition of visual objects, particularly for faces (Quiroga, 2016; Hawkins et al., 2017). Despite the absence of a laminar neocortex, birds also exhibit a remarkable ability to perceive and recognise the faces of conspecifics (Watanabe and Ito, 1990; Nakamura et al., 2003; Patton et al., 2010) and humans (Soto and Wasserman, 2011; Marzluff et al., 2012) across transformations in lighting, distance and viewpoint. The neuronal mechanisms by which visual categories are represented in the avian visual system is currently undetermined, but likely arises from circuitry that is homologous with associative cell-types found in the mammalian extrastriate visual cortex (Atoji and Karim, 2014; Briscoe et al., 2018).

In mammals, incoming visual information from primary visual areas is processed hierarchically along a ventral (‘what’) pathway that terminates in an extrastriate region known as the anterior inferior-temporal (IT) cortex (Ungerleider and Haxby, 1994; Freiwald and Tsao, 2010). Increasingly complex and view invariant representations of biologically relevant object categories, such as scenes (Kornblith et al., 2013), body parts (Pinsk et al., 2009), and faces (Tsao et al., 2003; Freiwald and Tsao, 2010) emerge in extrastriate cortex. For example, electrophysiological recordings from six functionally connected regions (known as ‘face-patches’) of macaque extrastriate cortex contain populations of neurons that respond with extreme selectively to faces (Tsao et al., 2003; Tsao et al., 2006; Moeller et al., 2017). Face-selective neurons in posterior ‘face-patches’ represent low-level dimensions of facial features in specific viewpoints (Freiwald et al., 2009; Issa and DiCarlo, 2012), whereas face-selective neurons in the most anterior ‘face-patch’ represent more complex dimensions of facial features across any viewpoint (Freiwald and Tsao, 2010; Chang and Tsao, 2017). Thus, the macaque ‘face-patch’ system generates a highly view-invariant 3-D representation of facial identity.

A wealth of neurobiological evidence suggests that pigeons also process visual (‘what’) information hierarchically (Nguyen et al., 2004; Stacho et al., 2016), and that despite a nuclear architecture, the functional connectivity of the pigeon visual system is astoundingly similar to the macaque visual system (De Groof et al., 2013; Shanahan et al., 2013). Like mammals, birds have two visual pathways. The thalamofugal pathway, homologous to the mammalian geniculo-striate pathway, projects from the retina to the principal optic nuclei and then to the visual wulst (Hodos and Karten, 1970; Shimizu and Bowers, 1999). The tectofugal visual pathway, in contrast, is homologous with the mammalian colliculo-pulvinar-cortical pathway, and travels from the retina to the optic tectum and then to the nucleus rotundus (nRT) of the thalamus, then to a primary visual structure known as the entopallium (ENTO), and finally to three visual association areas: the mesopallium ventrolaterale (MVL), nidopallium frontolaterale (NFL), and area temporo-parieto-occipitalis (TPO) (Husband and Shimizu, 1999; Krützfeldt and Wild, 2005).

Surprisingly, only a single study (Scarf et al., 2016) has investigated whether neurons in ENTO exhibit a category level representation of faces, but failed to demonstrate the presence of any face-selective neurons. In fact, very little is known about the response properties of neurons downstream of ENTO in NFL, MVL, and TPO, but preliminary recordings from NFL (Johnston et al., 2017) and MVL (Azizi et al., 2019) suggest that these association structures are functionally homologous with the mammalian extrastriate visual cortex. To determine if a ‘face-patch’ system also exists in the avian brain, it is necessary to understand how neurons in the association regions of the avian visual forebrain respond to complex visual categories. We performed bilateral electrophysiological recordings from ENTO, NFL, MVL, and TPO of freely moving pigeons during a Go/No-Go task that required them to discriminate between two stimulus sets consisting mainly of images depicting the face-region of two different pigeons. We hypothesised that neurons would respond selectively to images of faces, indicating biologically relevant percepts are encoded by neurons in the visual forebrain of birds.

## Materials and Methods

### Subjects

Thirteen pigeons (*Columba livia*) with previous experience on visual delayed-matching-to-sample tasks served as experimental subjects. The pigeons were housed in a colony room maintained at 20°C. Each pigeon had *ad libitum* access to grit and water and were fed a combination of wheat, peas, and corn. All 13 pigeons were maintained at approximately 85% of their free-feeding weight and individually housed for the duration of the experiment. All experimental procedures were approved by the University of Otago Animal Ethics Committee and conducted in accordance with the University of Otago’s Code of Ethical Conduct for the Manipulation of Animals.

### Apparatus

Pigeons were trained and tested in standard operant boxes measuring 36 cm wide, 32.5 cm high, and 34.5 cm deep. Stimuli were presented on a 17-inch monitor set at a resolution of 1284 × 1024. Situated in front of the monitor was a Carroll Touch infrared touch frame (EloTouch, baud rate 9600, transmission time 20 ms) that registered the XY coordinates of all the pecks. To prevent accidental activation of the touch frame by the pigeon’s body, a transparent Perspex panel with a single square opening (2.5 cm × 2.5 cm) was placed in front of the touch frame. Stimuli were presented on the monitor and the pigeons were required to peck at the image. A food hopper was positioned underneath the floor directly in front of the centre of the screen and 110 mm below the lower centre hole.

### Stimuli

**FIGURE 1:**
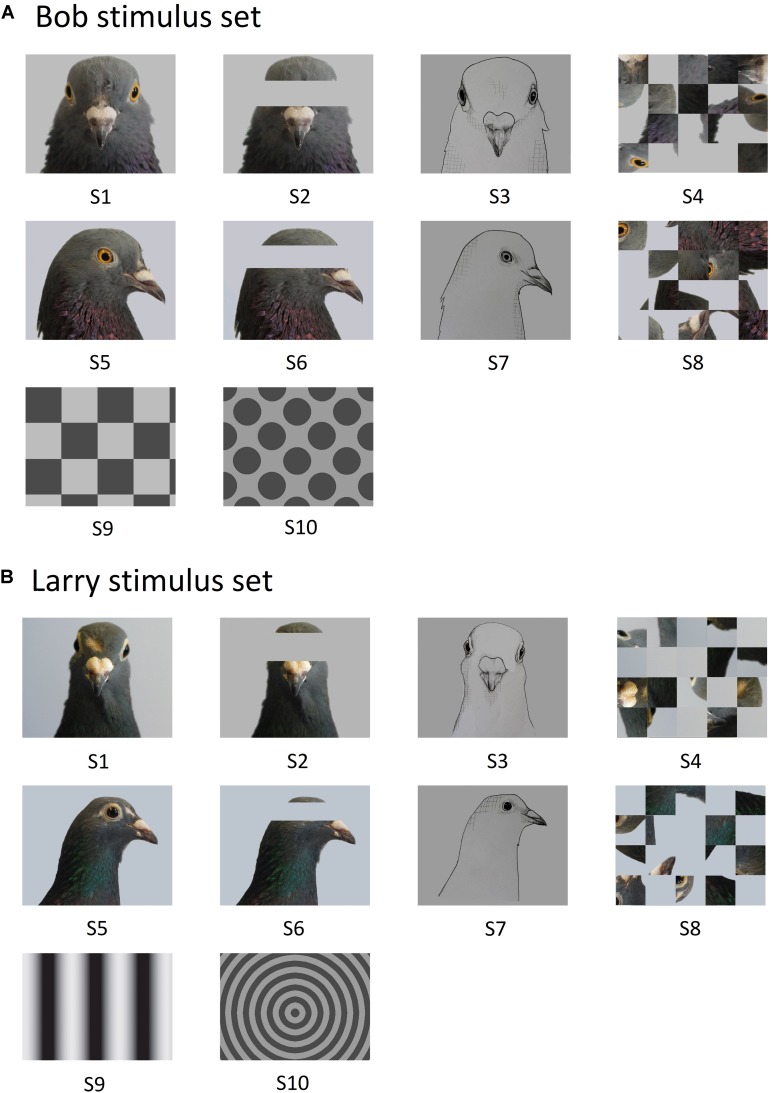
The 10 stimuli comprising image set Bob **(A)** and Larry **(B)**. Portrait face (S1), portrait face, eyes occluded (S2), portrait face, minimalistic line drawing (S3), portrait face, scrambled (S4), profile face (S5), profile face, eyes occluded (S6), profile face, minimalistic line drawing (S7), profile face, scrambled (S8), checkerboard geometric pattern or sine grating geometric pattern (S9), and spots geometric pattern or concentric circles geometric pattern (S10).

Twenty different images were used as stimuli and were taken using a Cannon DS126291 (12.2 Megapixel) digital camera and edited using Paint Shop Pro (Version 7) computer software. The colour pallets of each stimulus were approximately matched to ensure consistent brightness. Each set contained images of two different pigeon’s faces, hereafter referred to as Bob and Larry. The set of 10 Bob stimuli was composed of: a portrait face, portrait face (eyes occluded), portrait face (minimalistic line drawing), portrait face (scrambled), profile face, profile face (eyes occluded), profile face (minimalistic line drawing), profile face (scrambled), checkerboard geometric pattern and spots geometric pattern (see Figure 1A). The set of 10 Larry stimuli was composed of: a portrait face, portrait face (eyes occluded), portrait face (minimalistic line drawing), portrait face (scrambled), profile face, profile face (eyes occluded), profile face (minimalistic line drawing), profile face (scrambled), sine grating geometric pattern and concentric circle geometric pattern (see Figure 1B). All stimuli were presented against a black background.

### Behavioural Task

**FIGURE 2:**
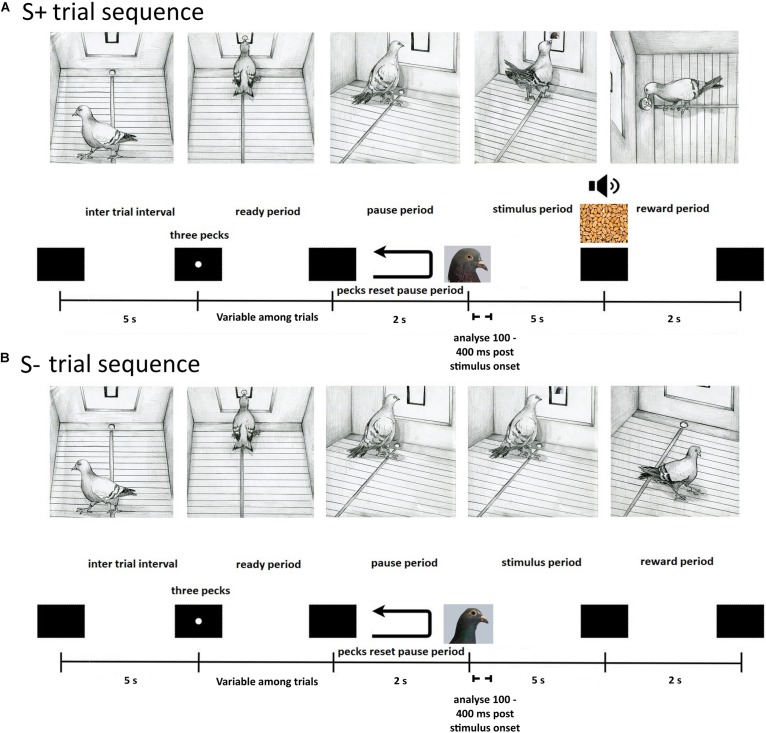
Depiction of the sequence of events within a single experimental trial for S+ **(A)** and S– **(B)** stimuli. **(A)** S+ trials began with a black screen during the inter trial interval, lasting 5 s, after which an orienting stimulus appeared. The pigeon was then required to peck the orienting stimulus three times during the ready period to initiate a 2 s pause period with a black screen. An S+ stimulus was then displayed within the central response key on the screen for a minimum of 5 s during the stimulus period. A peck was required to deliver a grain reward from the hopper (2 s) that was paired with a tone cue (1000-Hz) and illumination of the hopper, followed by the inter trial interval. On unrewarded S+ trials, an equivalent 2 s period was paired with a tone cue (1000-Hz) and illumination of the hopper, letting pigeons know they responded correctly. **(B)** S– trials began with a black screen during the inter trial interval, lasting 5 s, after which an orienting stimulus appeared. The pigeon was then required to peck the orienting stimulus three times during the ready period to initiate a 2 s pause period with a black screen. An S– stimulus was then displayed within the central response key on the screen for 5 s during the stimulus period, followed by the inter trial interval.

The pigeons were initially trained to eat grain from the food hopper. Next they were autoshaped to respond to a white dot three times to receive a reward. The pigeons were then trained on a Go/No-Go task to discriminate between the Bob and Larry stimuli. The procedure on a typical trial was as follows (see Figure 2). At the end of a 5 s inter trial interval (ITI), an orienting stimulus (white dot) was displayed. Three pecks to the orienting stimulus turned it off and initiated a 2 s pause period. Pecks during the pause period extended the pause period duration by 2 s. Following the pause period, either a Bob or Larry stimulus was displayed for at least 5 s. During trials in which an S+ stimulus was displayed, the first peck after 5 s resulted in 2 s access to grain, accompanied by a 1000-Hz tone and the illumination of the hopper. On S- trials, the stimulus turned off after the 5 s presentation period.

A session consisted of 240 trials, taking approximately 1 h and 20 min to complete, with each of the 20 images presented 12 times in a random order. Due to the large number of trials, correct S+ trials were rewarded approximately 70% of the time. Unrewarded S+ trials also consisted of a 2 s period during which the 1000-Hz tone was played and the hopper light was illuminated, but no reward was delivered. Discrimination training ended when the subjects attained a discrimination ratio (DR) of 0.75 in a session. The DR was calculated as the number of responses to the S+ stimuli divided by the number of responses to both the S+ and S- stimuli. Responses were accumulated only during the 5 s presentation period. Upon reaching criterion, pigeons were implanted with a movable microdrive. Seven birds were trained with image set Bob as the S+ stimuli (M5, M20, K1, K18, K22, Z11, and Bev), while for the other seven the image set Larry served as the S+ stimuli (M14, Z28, K11, K21, Z19, Z28, and Q42).

### Surgery

Once the pigeons were reliably completing the task with at least a DR of 0.75, stereotaxic surgery was performed to install a movable microdrive into the target brain area (Bilkey et al., 2003). A mixture of Ketamine (30 mg/kg) and Xylazine (6 mg/kg) was injected into the pigeon’s legs as an anaesthetic. The feathers on the head were then removed. The pigeons were placed in a Revzin stereotaxic adapter (Karten and Hodos, 1967) to immobilise the head and a topical anaesthetic (10% Xylocaine) was applied to the scalp. The skin overlying the skull was retracted exposing the skull, and six stainless steel screws were inserted into the skull. One of these screws served as the ground screw. A hole was drilled above the targeted area, as defined by Karten and Hodos (1967), and the dura was removed. A microdrive housing the electrodes was lowered into the hole until the tips of the electrodes were positioned above ENTO, NFL, MVL, and TPO. The microdrive was then secured to the skull using dental acrylic, and the wound was sutured closed. Xylocaine was applied again before the pigeons were placed into a padded and heated recovery cage. The pigeon remained in the recovery cage until it had returned to an active state, and then returned to their home cage where they were given another 7 days to recover before experimental sessions began.

### Neuronal Recording

The microdrives housed eight 25 μm Formvar-coated nichrome wires (California Fine Wire, Grover Beach, CA, United States) used to measure single neuron activity. For each experimental session we searched for activity on any one of the eight wires and used one of the remaining wires as the indifferent. The signals were amplified (x10000) using a GrassP511K amplifier (Grass Instruments, Quincy, MA, United States) and 50 Hz noise was eliminated. A CED (Cambridge Electronic Design, Cambridge, United Kingdom) electrophysiology system with Spike2 software stored and analysed the data. Cells were isolated using CED’s template matching capacity (thereby eliminating artefacts) sampling at a rate of 20000 Hz. The only selection criterion was that the isolated neuron had a signal-to-noise ratio of no less than 2:1. A separate computer controlled the behavioural task and sent codes to the CED system to align key task events. Following each recording session, the electrodes were advanced approximately 40 μm before the pigeon was returned to their home cage. If we did not record from any neural activity the electrodes were moved approximately 20 μm, and the animal was returned to its cage. Recording sessions took approximately 1 h to complete. Pigeons completed one session daily for 5 days a week.

### Neural Analysis

All of the data for each neuron was loaded into MATLAB (version R2016B) for data analysis using custom written MATLAB code. Neurons were required to exhibit mean firing rates > 0.2 Hz during the ITI period across an entire experimental session to be included in the analysis. To be included for further analysis, a neuron had to be visually responsive, firing at a significant level (Paired *t*-test: modified Bonferroni, *p* < 0.02; Keppel and Zedeck, 1982) to at least one of the 10 S+ stimuli relative to the baseline ITI activity. We next compared each recorded neuron’s baseline ITI mean firing rates with the mean firing rate elicited by the 10 S+ stimuli during the stimulus period (100–400 ms post stimulus onset) over the 12 stimulus presentations. We performed a comprehensive series of analysis steps to investigate the possibility that activity reflective of stimuli may be present in the first 100 ms of the Stimulus period. We calculated all neurons firing rates for every trial during the first two bins (50 ms bin size) of the stimulus period, and an equivalent two bin window in the middle of the ITI. *T*-tests between the first 100 ms of the stimulus period and ITI window showed that 19/405 (4.96%: uncorrected) and only 1/405 (0.24%: Holm–Bonferroni, *p* < 0.0001) total neurons with a strict Bonferroni correction showed significantly different firing rates between these periods. Therefore, while these analyses suggest that activity reflective of stimuli emerges approximately 100 ms post stimulus, it is important for future studies to consider that such representations may emerge at a slightly earlier time course in some structures of the tectofugal visual pathway.

We used custom written MATLAB code to find trials where the difference between the time of stimulus onset and the first peck made to the stimulus was <400 ms and excluded these trials from further analysis. Finally, each visually responsive neuron was classified as either excitatory (firing more to the onset of the stimulus than the baseline ITI level) or inhibitory (firing less to the onset of the stimulus than the baseline ITI level). Lastly, we assigned neurons a classification (excitatory or inhibitory) based on a binomial probability distribution of the total number of excitatory and inhibitory responses relative to the total number of stimuli that the neuron fired to at a significant level. We compared the total numbers of visually responsive versus non-visually responsive, and excitatory versus inhibitory cells between each region, hemisphere, and anteroposterior position. Each of these comparisons was performed by either using a Chi-Squared test (Holm–Bonferroni, *p* < 0.008), or a Fishers Exact test (Holm–Bonferroni, *p* < 0.005) if the frequency of sampled neurons in a group was <5. We used a total of 6 Chi-squared tests and 9 Fishers Exact tests for these comparisons.

### Stimulus Selectivity

Once we determined that a neuron was visually responsive as per the previous criteria, we next assessed whether any neurons exhibited selective responses to faces such as those found in the category-selective ‘face-patches’ in macaque extrastriate cortex (Freiwald et al., 2009; Freiwald and Tsao, 2010). We also determined if neurons responded selectively only to geometric stimuli, indicating that these neurons may be encoding low-level features of our visual stimuli (Koenen et al., 2016). The selectivity patterns of isolated neurons’ responses to the 10 S+ stimuli (either Bob or Larry) during the ITI and stimulus period were determined using a series of 10 paired *t*-tests with a modified Bonferroni correction (conservative alpha of *p* < 0.02; Keppel and Zedeck, 1982).

We classified neurons as face-selective according to six possible classifications (see Table 1) based on how the neuron responded to the S+ stimuli. The first three classifications involved significant responses only to the intact face images. We classified neurons as face-selective (F) based on the following criteria. Neurons that responded significantly to only S1 (portrait face) were considered viewpoint selective, and were categorised as F-1 selective, those that fired at a significant level only to S5 (profile face) were also considered viewpoint selective and were categorised as F-2 selective, and those that fired at a significant level to S1 (portrait face) and S5 (profile face) were considered viewpoint invariant and were classified as F-3 selective.

**Table 1:** Classification of face-selective and geometric-selective neurons.

Face-selective			Geometric-selective		
	
F-1	F-2	F-3	G-1	G-2	G-3
Fired at a significant level to S1	Fired at a significant level to S5	Fired at a significant level to S1 and S5	Fired at a significant level to S9	Fired at a significant level to S10	Fired at a significant level to S9 and S10
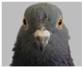	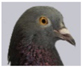	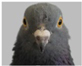	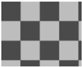	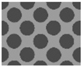	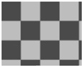
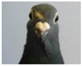	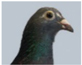	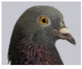	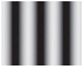	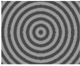	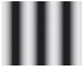
		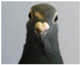			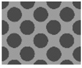
		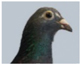			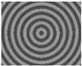


The second three classifications involved significant responses to the geometric stimuli (see Table 1). We classified neurons as geometric-selective based on the following criteria. Neurons that fired at a significant level to S9 (checkerboard or sine grating) and no other stimulus were classified as G-1 selective, those that fired at a significant level to S10 (spots or concentric circle) and no other stimuli were classified as G-2 selective, and those that fired at a significant level only to S9 (checkerboard or sine grating) and Stim 10 (spots or concentric circle) were categorised as G-3 selective.

### Stimulus Selectivity: Population Analysis

The mammalian ventral visual stream is organised in a series of hierarchical processing stages that encode increasingly explicit information on object identity and category (Kriegeskorte et al., 2008b; Yamins et al., 2014). Therefore, viewpoint invariant representations of object categories (e.g., faces and animals) can be extracted at the level of a neuronal population in IT cortex, but not from a population at lower stages of the visual hierarchy (e.g., V1/V2; Yamins et al., 2014). As described in Kriegeskorte et al. (2008a), Representational Similarity Matrices (RSM) are a tool used to extract categorical representations by comparing a correlate of neural activity (in our case the population firing rates) associated with each pair of visual stimulus conditions between multiple brain regions. An RSM is composed of a symmetric matrix of cells that each contain a number reflecting the similarity/dissimilarity of firing rates between each pair of stimulus conditions averaged across a neuronal population. The similarity of stimulus conditions is measured as correlation distance (R) over space (1 for total correlation, 0 for no correlation, and -1 for total anticorrelation). When stimuli are organised by category along the rows of the matrix, the resulting RSM reflects the position of each stimulus in the high-dimensional space of the neuronal population.

Computational modelling suggests that the pigeon visual system is organised in a hierarchical feed-forward progression (Soto and Wasserman, 2012), and electrophysiological recordings indicate that category level information may be represented at a population level in association visual forebrain regions (Azizi et al., 2019). We therefore generated an RSM for the ENTO, MVL, NFL, and TPO to investigate whether any of these regions exhibited a population coding of conspecific’s faces as a perceptual category. Neuronal data during the stimulus period (100–400 ms post stimulus onset) on S+ and S- trials was used for the RSM analysis. The RSM stimulus period included S- trial data, and therefore differed from that used for stimulus selectivity (only S+ trial data) so that the RSM reflected each isolated neuron’s responses to the entire stimulus set. We then computed Pearson correlation coefficients (Pearson’s *R*) of the average firing rate during the stimulus period, for each stimulus, with the average firing rates elicited by all other stimuli. These correlation coefficients were used to generate an RSM for each neuron that was stored in a 3-D array. Individual neuron’s Pearson correlations were then averaged together to create an RSM for ENTO (*n* = 65), MVL (*n* = 37), NFL (*n* = 28), and TPO (*n* = 12).

### Histology and Electrode Track Reconstruction

When the electrodes reached the end of ENTO, NFL, MVL, or TPO, the final recording position was marked by sending a 15 mA (9V) current through each electrode for 10 s to create an electrolytic lesion at the tip of each electrode. The pigeons were then anaesthetised deeply with isoflurane and perfused with physiological saline and 10% formalin. The brains were removed and kept for five days in 10% formalin in 30% sucrose. They were then frozen and cut into 40 μm sections. Cresyl violet was used to stain every 5th section of the brain. The position of the recorded neurons was located by using the position of the electrolytic lesion, track reconstructions, and depth records.

## Results

### Electrode Positions

**FIGURE 3:**
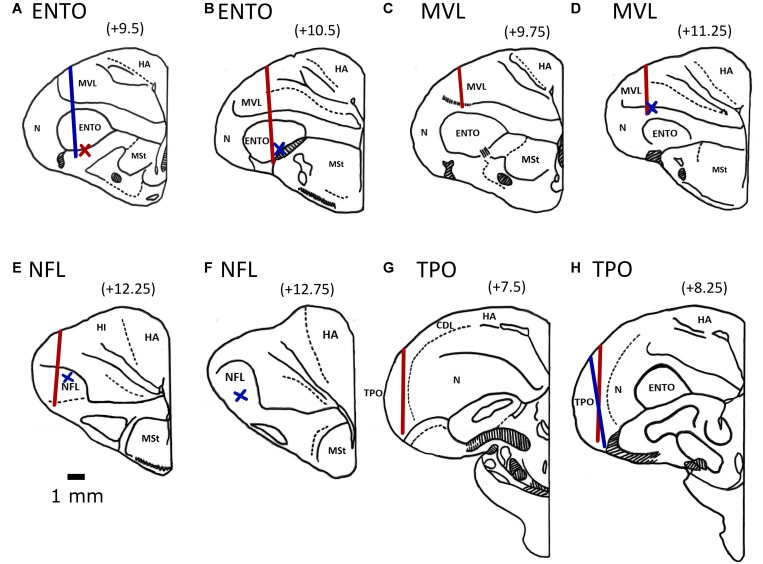
Electrode track position reconstructions for the two ENTO birds at AP + 9.5 **(A)** and AP + 10.5 **(B)**, the MVL bird at AP 9.75 **(C)** and two birds at AP + 11.25 **(D)**, two NFL birds at AP + 12.25 **(E)** and one bird implanted at AP + 12.75 **(F)**, the TPO bird implanted at AP + 7.5 **(G)** and two birds implanted at AP + 8.25 **(H)**. Both left and right electrode tracks are displayed on the same section. Tracks for birds with implants in the left hemisphere are displayed by the blue line and for birds with implants in the right hemisphere by a red line. We determined the termination point of electrode tracks that were unable to be recovered based on depth records. Termination points for birds with implants in the left hemisphere are displayed by a blue cross and for birds with implants in the right hemisphere by a red cross. All electrodes were advanced to the top of the intended region before recording began. The following are the brain regions as defined by Reiner et al. (2004): MSt, medial striatum; HA, hyperpallium apicale; HD, hyperpallium denso-cellulare; HI, hyperpallium intercalatum; Hp, hippocampus; MVL, mesopallium ventrolateral; N, nidopallium; NFL, nidopallium frontolaterale.

All electrode tracks were within the borders of the targeted ENTO, NFL, MVL, and TPO regions (Karten and Hodos, 1967; Stacho et al., 2016). The histological track placements are shown in Figure 3. For ENTO, two pigeons (M14 and M20) had microdrives installed at positions AP ± 9.5, and ML ± 6.0, and two pigeons (M5 and Z26) at positions AP ± 10.5 and ML ± 6.0. For MVL, two pigeons (Z11 and Z19) had microdrives installed at positions AP ± 9.75 and ML ± 6.7, and two pigeons (K22 and K25) at positions AP ± 11.25 and ML ± 6.7. For NFL, two pigeons (K1 and K11) had microdrives installed at positions AP ± 12.25 and ML ± 6.0, and two pigeons (K18 and K21) at positions AP ± 12.75 and ML ± 6.3. For TPO, one pigeon (Q42) had a microdrive installed at position AP ± 9.5 and ML ± 8.8, and two pigeons (Z28 and BEV) AP ± 8.25 and ML ± 9.5. Although for several birds we were unable to recover the electrode track placements, all recovered track locations were within ± 0.6 mm of their intended AP and ML implant co-ordinates (see Figure 3).

### Behavioural Performance

The discrimination ratio (DR) was calculated from the number of correct pecks to the S+ stimuli divided by the total number of S+ and S- pecks. We then averaged the DR across the total number of sessions each bird completed to find its mean DR. All 13 birds achieved a high level of performance, exhibiting DRs of 96% (M14), 94% (M5), 92% (Z26), 85% (M20), 88% (K1), 88% (K11), 94% (K18), 97% (K22), 91% (Z11), 92% (Z19), 83% (Z28), 96% (Q42), and 84% (Bev) correct responses. There was no difference in the DRs for birds whose S+ stimulus was the Bob set (92%) compared to birds whose S+ stimulus was the Larry set (88%), [Paired *t*-test, *t* = 1.16 (6), *p* = 0.30]. We next compared all birds’ DR performance for the face images (95%) with the four line-drawn face images (79%), occluded-eyes face images (95%), and scrambled face images (96%) across all sessions. All birds DR performance was significantly lower for the four line-drawn stimuli compared with the four intact face stimuli [Paired *t*-test, *t* = 4.54 (12), *p* = <0.001]. There were no significant differences between birds’ DR performance for the four intact face images compared with the four face images with occluded-eyes [Paired *t*-test, *t* = 0.025 (12), *p* = 0.74], and the four scrambled face images [Paired *t*-test, *t* = 0.02 (12), *p* = 0.40].

### Basic Response Properties of ENTO, MVL, NFL, and TPO

All four regions exhibited a large proportion of neurons that were visually responsive to at least one of the S+ stimuli, verifying that each region is heavily involved in processing tectofugal visual information (see Table 2). Although visual responsivity was greatest in MVL (40%), followed by ENTO (37%), NFL (32%), and TPO (23%), a Chi-squared test revealed no significant differences in the relative numbers of visually responsive neurons between the four areas [χ^2^(3) = 3.72, *p* = 0.29].

**Table 2:** Proportion of visually responsive, excitatory and inhibitory neurons in ENTO, MVL, NFL, and TPO.

Region	Visually responsive	Excitatory	Inhibitory
ENTO	65/176 (37%)	38/65 (58%)	27/65 (42%)
MVL	37/93 (40%)	22/37(60%)	15/37(40%)
NFL	28/88 (32%)	13/28 (46%)	15/28 (54%)
TPO	12/48 (25%)	0/12 (0%)	12/12 (100%)


The percentages of cells in each of the four areas that displayed excitatory or inhibitory activity is also shown in Table 2. Both ENTO and MVL exhibited a greater proportion of excitatory neurons than inhibitory neurons, whereas both NFL and TPO displayed the opposite trend. There were no significant differences in the relative numbers of excitatory and inhibitory neurons isolated in ENTO (Fishers Exact test: *p* = 0.08), MVL (Fishers Exact test: *p* = 0.42), or NFL (Fishers Exact test: *p* = 0.82). All visually responsive neurons isolated in TPO were inhibitory.

### Hemispheric Comparisons Between ENTO, NFL, MVL, and TPO

Previous ENTO studies have demonstrated that pigeons exhibit significantly greater visually responsive neurons in the left hemisphere compared to the right hemisphere (Verhaal et al., 2012). In contrast, although far fewer studies have been conducted, there is little evidence of lateralisation in MVL, NFL, and TPO (Stacho et al., 2016). We compared the characteristics of visually responsive neurons between the left hemisphere and right hemisphere of ENTO, MVL, NFL, and TPO to evaluate any possible differences in visual function between hemispheres. The results are shown in Table 3.

**Table 3:** Proportion of visually responsive, excitatory, and inhibitory neurons in the left and right hemisphere of ENTO, MVL, NFL, and TPO.

Region	Left overall	Right overall	Left excitatory	Right excitatory	Left inhibitory	Right inhibitory
ENTO	39/95 (41%)	26/81 (32%)	24/39 (62%)	14/26 (53%)	15/39 (38%)	12/26 (47%)
MVL	10/35 (29%)	27/58 (47%)	3/10 (30%)	19/27 (70%)	7/10 (70%)	8/27 (30%)
NFL	11/49 (22%)	17/39 (44%)	11/11 (100%)	2/17 (12%)	0/11 (0%)	15/17 (88%)
TPO	11/34 (32%)	1/14 (7%)	0/11 (0%)	0/1 (0%)	11/11 (100%)	1/1 (100%)


ENTO and TPO exhibited a greater proportion of visually responsive neurons in the left hemisphere relative to the right hemisphere. In contrast, MVL and NFL showed a greater number of visually responsive neurons in the right hemisphere compared with the left hemisphere. No significant differences in the number of visually responsive neurons were found between the right and left hemisphere of ENTO (Fishers Exact test: *p* = 0.27), MVL (Fishers Exact test: *p* = 0.12), NFL (Fishers Exact test: *p* = 0.04), or TPO (Fishers Exact test: *p* = 0.08).

We also compared the total number of visually responsive neurons classified as excitatory and inhibitory across the left and right hemisphere of ENTO, NFL, MVL, and TPO. ENTO and NFL exhibited a greater proportion of excitatory neurons in the left hemisphere than in the right hemisphere, and a greater proportion of inhibitory neurons in the right hemisphere compared with the left hemisphere. MVL exhibited a greater proportion of excitatory neurons in the right hemisphere relative to the left hemisphere, and a greater proportion of inhibitory neurons in the left hemisphere compared with the right hemisphere. No excitatory neurons were isolated in TPO, and we found that most of the inhibitory neurons were in the left hemisphere compared with the right hemisphere. We found a significant difference in the proportion of excitatory and inhibitory neurons between the left and right hemisphere of MVL [Chi Squared test: χ^2^(3) = 12.163, *p* = <0.007], and NFL [Chi Squared test: χ^2^(3) = 41.88, *p* = <0.0000001] but not ENTO [Chi Squared test: χ^2^(3) = 4.462, *p* = 0.21].

### Comparisons Between Anterior–Posterior ENTO and MVL

We also compared the characteristics of visually responsive neurons between the anterior and posterior regions of ENTO and MVL to examine any potential differences in visual processing. Insufficient numbers of visually responsive neurons were isolated at the anterior aspect of NFL (AP 12.75; *n* = 3) and posterior aspect of TPO (AP 7.5; *n* = 3) for functional comparisons to be made. The results are shown in Table 4.

**Table 4:** Proportion of visually responsive, excitatory, and inhibitory neurons in anterior and posterior regions in ENTO and MVL.

Region	Anterior	Posterior	Anterior excitatory	Posterior excitatory	Anterior inhibitory	Posterior inhibitory
ENTO (AP 9.5 vs. AP 10.5)	13/66 (20%)	52/110 (47%)	8/13 (61%)	30/52 (58%)	5/13 (39%)	22/52 (42%)
MVL (AP 9.75 vs. AP 11.25)	11/23 (47%)	26/70 (37%)	9/11 (82%)	13/26 (50%)	2/11 (18%)	13/26 (50%)


The overall proportion of visually responsive neurons was greater in posterior relative to anterior ENTO, and this difference was significant (Fishers Exact test: *p* = <0.0001). In contrast, there was little evidence for a difference in the number of visually responsive neurons isolated from anterior relative to posterior MVL (Fishers Exact test: *p* = 0.46).

We also compared the total numbers of visually responsive cells classified as excitatory and inhibitory across the anterior and posterior aspect of ENTO and MVL. ENTO and MVL exhibited a greater proportion of excitatory neurons in the anterior compared with posterior regions. In contrast, the posterior ENTO and MVL showed a greater proportion of inhibitory neurons compared with anterior regions. We found no significant differences in the proportion of excitatory and inhibitory neurons between anterior and posterior ENTO [Chi Squared test: χ^2^(3) = 3.84, *p* = 0.27], and MVL [Chi Squared test: χ^2^(3) = 8.909, *p* = 0.03].

### Stimulus Selectivity

We assessed the stimulus selectivity exhibited by visually responsive neurons in ENTO, MVL, NFL, and TPO and the results are show in Table 5.

**Table 5:** Number of face-selective and geometric-selective neurons in ENTO, MVL, NFL, and TPO.

			Face-selective			Geometric-selective		
				
	Total face-selective	Total geometric-selective	F-1	F-2	F-3	G-1	G-2	G-3
ENTO	10/65 (15%)	5/65 (8%)	7	2	1	2	2	1
MVL	2/37 (5%)	3/37 (8%)	0	2	0	1	2	0
NFL	2/28 (7%)	3/28 (11%)	1	1	0	1	2	0
TPO	1/11 (9%)	2/11 (18%)	0	1	0	0	2	0


**FIGURE 4:**
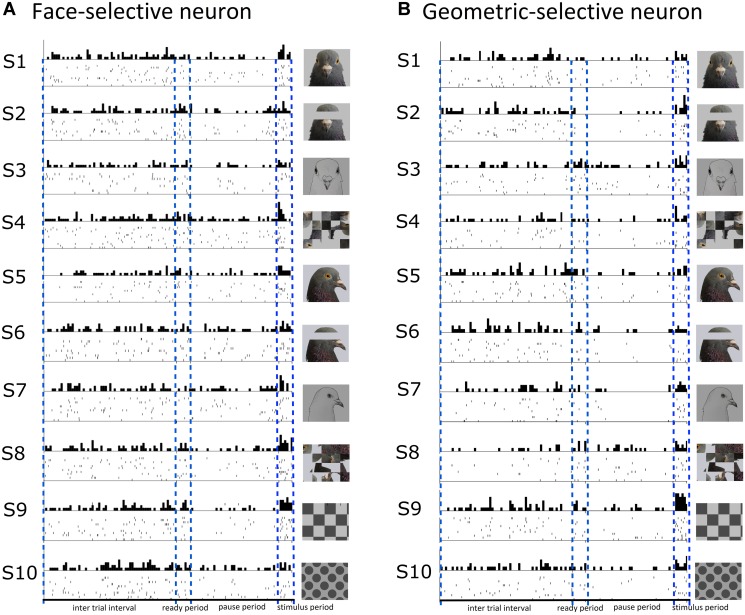
Raster and histogram plots of a neuron classified as face-selective **(A)** and geometric-selective **(B)**. **(A)** Example of a neuron in the posterior entopallium that exhibited excitatory firing at a significant level to the stimulus S1 (portrait face) relative to the ITI, but also responded to the other stimuli during the stimulus period. **(B)** A neuron in the posterior entopallium that fired in an excitatory manner at a significant level to a geometric stimulus S9 (checkerboard) relative to the ITI.

A total of 15/141 (11%) visually responsive neurons isolated across ENTO, MVL, NFL, and TPO were classified as face-selective. Most of the face-selective neurons were found in ENTO and the majority (10/15; 67%) of these face-selective cells responded to the portrait face image. Fewer numbers of face-selective neurons were found in MVL, NFL, and TPO and fired either to portrait (F-1) or profile (F-2) face stimuli, but not to both face images (F-3, see Table 5). While the 15 face-selective neurons fired at significant levels only to faces, they did not exhibit the selectivity of that shown by face-selective neurons in macaque extrastriate cortex (Tsao et al., 2006) and also responded to other visual stimuli (see Figure 4A for an example cell). Therefore, the neurons we classified as “face-selective” are not homologous with face-selective neurons in macaques, and their exact contribution to vision remains undetermined.

A total of 13/141 (9%) visually responsive neurons isolated across ENTO, MVL, NFL, and TPO responded selectively to geometric stimuli and were classified as geometric-selective (see Figure 4B for an example cell). Two of the geometric-selective neurons in ENTO and a single neuron in MVL and NFL only fired to the concentric circle or spots. Two of the geometric-selective neurons in all regions fired to either the checkerboard or gratings patterns. The low number of total neurons that we classified as “geometric-selective,” and small number of geometric stimulus manipulations means that we are unable to determine exactly what stimulus dimensions these cells responded to.

### Stimulus Selectivity: Population Analyses

**FIGURE 5:**
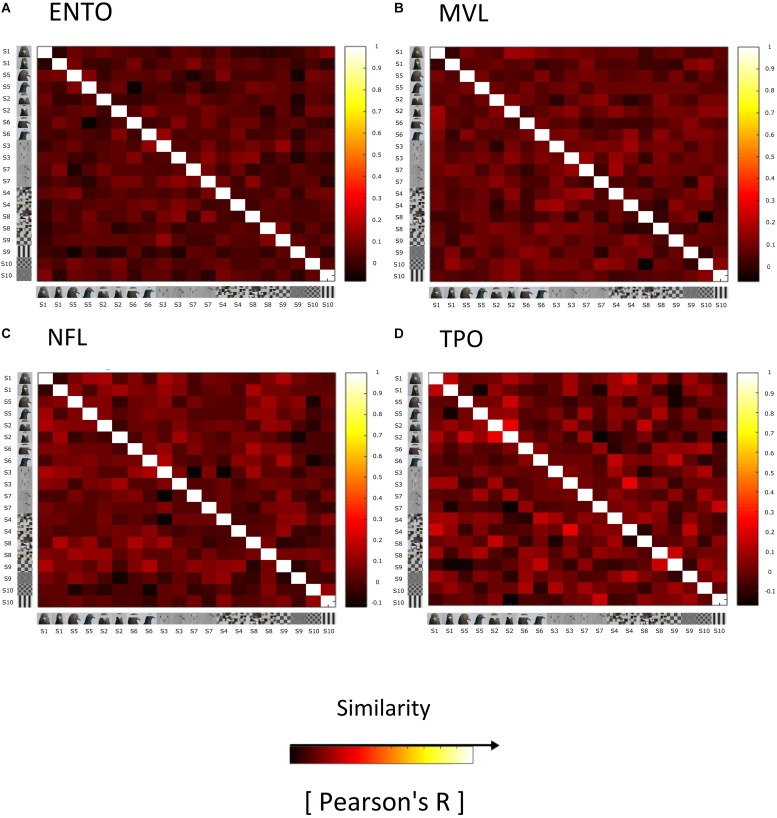
Representational similarity matrices (RSM) showing the similarity of responses elicited by the stimuli for all visually responsive neurons in **(A)** ENTO, *n* = 65, **(B)** MVL, *n* = 37, **(C)** NFL, *n* = 28, and **(D)** TPO, *n* = 11. The similarity of responses for each stimulus is measured as Pearson correlation across space (Pearson’s *R*), averaged across all sessions for each region. The colour code reflects the similarity of spatial correlations (see colour bar; +1 being total similarity, 0 for no correlation, and –1 as total anticorrelation) computed separately for each RSM (for *R*-values and their histograms). The Bob and Larry examples of individual stimuli are organised into Face (S1, S5, S2, S6, S3, and S7) and Non-Face (S4, S8, S9, and S10) categories along the rows of the matrix. RSM analysis failed to demonstrate a clustering of strong correlations for faces relative to non-face stimuli in ENTO, MVL, NFL, or TPO, in contrast to the face-representation exhibited in monkey and human IT Cortex (Kriegeskorte et al., 2008b).

On the basis of single-unit analysis, we found no evidence for face-selective neurons in ENTO, MVL, NFL, and TPO. One possibility is that, as in primates, face-category information is represented at a population level in the pigeon visual forebrain. We therefore generated Representational Similarity Matrices (RSMs) for each of our targeted regions (ENTO, NFL, MVL, and TPO) in order to assess weather face-category information was represented at a population level. We observed no evidence of a clustering of similar correlations for face images in ENTO (Figure 5A), MVL (Figure 5B), NFL (Figure 5C), or TPO (Figure 5D) that was indicative of a categorical population representation like that found in primate extrastriate cortex (Kiani et al., 2007; Kriegeskorte et al., 2008b).

We verified that there was no population level representation of face-information in any region by comparing the relative differences in the normalised firing rates elicited by faces, eye-manipulations, scrambled faces and geometric stimuli between ENTO, NFL, MVL, and TPO. Relative differences in firing rates for each region were calculated by taking the mean of the normalised firing rates for each visually responsive neuron to faces, and subtracting the resulting values from the means of eye-manipulation, scrambled, and geometric stimuli. There were no significant differences in the mean firing rates for faces, compared to eye-manipulation [Kruskal–Wallis test (3): χ^2^(2) = 4.11, *p* = 0.249], scrambled [Kruskal–Wallis test (3): χ^2^(2) = 4.79, *p* = 0.187], and geometric stimuli [Kruskal–Wallis test (3): χ^2^(2) = 1.15, *p* = 0.765] between ENTO (*n* = 65), MVL (*n* = 37), NFL (*n* = 28), and TPO (*n* = 12).

## Discussion

We performed bilateral electrophysiological recordings from ENTO, NFL, MVL, and TPO of freely moving pigeons during a Go/No-Go task that required discriminating between two stimulus sets that included images of the faces of two different pigeons. ENTO, NFL, MVL, and TPO all exhibited a relatively large proportion of neurons (between 21 and 37%) responsive to at least one visual stimulus, verifying that each region is heavily involved in the appraisal of tectofugal visual information. In stark contrast to the human (Nasr and Tootell, 2012; Schalk et al., 2017), macaque (Tsao et al., 2006; Chang and Tsao, 2017), marmoset (Hung et al., 2015), and sheep (Kendrick and Baldwin, 1987) extrastriate visual cortex, very few neurons in the pigeon brain fired selectively to faces (15 of 407 total neurons: 4%). These 15 neurons did not respond to faces at levels comparable to mammalian face-selective neurons (Tsao et al., 2006) and therefore we do not consider them to be functionally homologous. The absence of face-selective neurons suggests that birds’ solution to the challenges of object representation may be mechanistically different to mammalian species, but can also be explained by divergences in birds’ visual physiology and behaviour in visual discrimination tasks.

### How do Birds Construct Visual Representations of Objects?

Birds are perhaps the most visually complex vertebrate species (Shimizu and Bowers, 1999; Jarvis et al., 2005) and like primates, possess exceptional foveal visual acuity for analysing object features (Tyrrell et al., 2014). Recent breakthroughs in our understanding of high-level vision in macaque IT cortex have shown that patches of stimulus-selective neurons emerge in a large swath of foveal extrastriate cortex from V4 through to anterior IT cortex. Stimulus-selective neurons can be selective to object class (e.g., faces: Tsao et al., 2003), or for other stimulus dimensions (e.g., curved or rectangular stimuli: Yue et al., 2014). Ethologically relevant visual percepts exhibit the greatest representation of stimulus-selective neurons in extrastriate cortex (Kriegeskorte et al., 2008b; Moeller et al., 2017). Electrophysiological recordings from the macaque ‘face-patch’ system have revealed that increasingly complex and view-invariant representations of visual objects are encoded by neurons in stimulus-selective patches (Freiwald and Tsao, 2010) and that the computational goal of such neurons is to measure the feature dimensions of visual objects (Chang and Tsao, 2017). If the hierarchical organisation of object representation demonstrated in macaque extrastriate cortex is conserved across vertebrate species, then birds may also exhibit clusters of category-selective neurons in association forebrain structures of the tectofugal visual pathway. While we found no evidence to suggest that face-selective neurons exist in ENTO, MVL, NFL, and TPO, our sampled neurons’ high responsivity to visual stimuli indicates that these regions may perform homologous functions to extrastriate visual cortex.

### Is the Avian Face a Socially Significant Stimulus for Birds?

A possible explanation for our inability to locate category-selective neurons is that the freely moving behavioural paradigm and close-proximity of the visual stimuli in an operant chamber inhibit the processing of faces as a global configuration of feature dimensions. While neurons in the pigeon visual forebrain exhibit large receptive fields like in extrastriate cortex (Kimberly et al., 1971; Xiao et al., 2006), pigeons preferentially attend to and analyse the local features of visual stimuli over their global configuration in visual discrimination tasks (Cavoto and Cook, 2001; Aust and Braunöder, 2015). Pigeons’ predisposition to discriminate between object categories based on local features is associated with a left-hemispheric dominance of the tectofugal visual pathway in visual discrimination and learning (Yamazaki et al., 2007; Xiao and Güntürkün, 2009; De Groof et al., 2013). Moreover, electrophysiological recordings from ENTO suggest that the association of visual stimuli with reward progressively increases the number of visually responsive neurons in the left hemisphere and modulates their capacity to differentiate between rewarded and unrewarded visual stimuli (Verhaal et al., 2012). As a result, top-down dopaminergic visuo-motor feedback via projections with the nidopallium caudolaterale (NCL) and motor structures may cause visually guided behaviour in birds to be increasingly driven by local perceptual cues to solve the visual discrimination over time (Schultz, 2016; Güntürkün et al., 2018). An important implication of birds’ local-precedence effect is that our pigeons may have learned to discriminate between stimulus sets by attending to a component feature, colour, or background patterns predictive of the S+ images. For instance, Cook et al. (2013) trained pigeons to discriminate between line drawings of birds and mammals and subsequently tested their transfer to novel instances with manipulated features. The authors discovered that pigeons discriminated between the two object classes by using the contrast between animal figures from the background and the orientation axis of the body, but not using features of the face region or body parts. Perhaps further compounding a bias toward local-feature based strategies is the fact that our pseudocategorisation task design required birds to discriminate between S+ and S- image sets without providing an incentive or sufficient viewpoint-invariant information to relate face stimuli to their corresponding real world representations.

For example, Peissig et al. (2019) demonstrated that pigeons’ categorisation performance was significantly impaired in a pseudocategorisation task that required birds to peck a key corresponding to one of four viewpoint rotations (-90, 0, 90, and 180°) of four different 3-D geometric objects, relative to a categorisation task that required birds to peck a single viewpoint rotation of only one of the same four objects. Therefore, pigeons in the Pseudocategorisation group only responded based on perceptual groupings of local orientation-dependant features that did not correspond to object identity, whereas the Categorisation group achieved highly accurate responding with access to viewpoint-invariant information on a particular object’s identity. Our pigeons’ ability to discriminate between S+ and S- line-drawn images of faces was significantly impaired relative to face, scrambled, eye-manipulations, and geometric stimuli. While many of the visual cues indicative of a bird’s face were still present in the line drawings, the form of these images was likely an insufficient perceptual cue to relate to the other rewarded face exemplars during discrimination. Therefore, while primates can easily perceive and recognise line-drawn images of faces (Freiwald et al., 2009), birds appear to reduce the stimulus space to a few key dimensions in a pseudocategorisation discrimination paradigm and do not see the correspondence between face drawings, or other sub-categories within the stimulus set and their real world representations. A strategy based on the analysis of local visual features can explain why all 13 of our pigeons were able to learn successfully to discriminate between the S+ and S- stimuli, but did not facilitate the visual perception of face stimuli versus non-face stimuli. As a result, our electrophysiological recordings may be more reflective of the reward prediction or preparatory motor act of pecking, as opposed to the perception of stimuli as examples of face and non-face object categories.

Other lines of evidence indicate that faces do hold relevance for birds. For example, neurobiological and behavioural studies in chicks (Vallortigara, 1992; Vallortigara and Andrew, 1994; Mayer et al., 2016) indicate that the avian right hemisphere plays a critical role in both the perception and recognition of conspecifics as a global configuration of features. Moreover, both pigeons (Patton et al., 2010) and Japanese quail (Domjan and Nash, 1988; Domjan et al., 1989) spontaneously elicit courtship behaviour and preferentially attend to the face region of live and static images of conspecifics indicating that they recognise the visual object as a potential mate. Determining the neural mechanism by which birds perceive and recognise conspecifics faces may require a task design that maximises attention to faces as a global combination of features without the psuedocategorisation of stimuli. An alternative Go/No-Go discrimination task would be one in which pigeons are trained to discriminate between an S+ and S- image set comprising images of two different pigeons faces, respectively, in various viewpoints (Watanabe and Ito, 1990). Pigeons can then be tested in a second phase to discriminate between examples of the original S+ and S- face stimuli that are all manipulated in the same way (e.g., all face stimuli scrambled). Another possible behavioural task design is to remove the S+/S- discrimination entirely and instead employ a passive fixation task in which individual stimuli belonging to separate object categories are all rewarded with grain (Azizi et al., 2019). To maximise attention to the stimuli, pigeons should be required to withhold pecking responses both during a pause period before stimuli are presented and after stimuli appear for a randomly determined duration (e.g., between 2 and 4 s), until a Go stimulus appears in place of the image.

Recent technological advances used in conjunction with the aforementioned behavioural tasks may further ensure that neuronal activity is reflective of visual stimuli. For instance, a system for tracking the gaze of laterally eyed vertebrates has recently been developed (Tyrrell et al., 2014), and could be used in conjunction with an S+/S- discrimination or passive fixation task while measuring the activity of neuronal populations across the tectofugal visual pathway with electrophysiological recordings or functional magnetic resonance imaging (fMRI: De Groof et al., 2013; Behroozi et al., 2017). The combination of such techniques may enable researchers to present visual stimuli at an appropriate distance to maximise global processing strategies and ensure that subjects view stimuli within their fovea, as is common practise in face perception studies using non-human primates (Tsao et al., 2003; Hung et al., 2015). However, another reason for why we were unable isolate a large proportion of face-selective cells may be that birds also depend on visual cues other than the face region to construct representations of socially significant objects. For example, birds lack the developed facial musculature of primates that is associated with dorsal face-selective patches for the processing of facial expressions and gaze direction in the superior temporal sulcus (STS) of macaques and humans (Moeller et al., 2008; Fisher and Freiwald, 2015). Indeed, there are also large patches of mammalian extrastriate cortex that measure the feature dimensions of body parts that are located directly adjacent to face-selective patches in macaque extrastriate cortex (Pinsk et al., 2009; Bao and Tsao, 2018).

### Functional Contributions of the Visual Forebrain to Object Recognition

Recent advances in avian neurobiology have revealed that birds’ dorsal telencephalon (comprising the dorsal ventricular ridge and Wulst) shares many genetic and functional similarities with mammalian neocortex, although it does not resemble the neocortex morphologically (Shimizu and Bowers, 1999; Briscoe et al., 2018; Gutiérrez-Ibáñez et al., 2018). For example, the avian dorsal telencephalon receives input from visual, auditory and somatosensory dorsal thalamic neurons, as does the neocortex (Reiner et al., 2004). Briscoe et al. (2018) used RNA sequencing to show that both the bird and crocodilian mesopallium regions share transcription factors regulating genes that control the development of associational neuronal types found in the mammalian neocortex (cells that forward projections only to other telencephalic targets). These cell types are classified as intra-telencephalic neurons (major excitatory cell types found in neocortical layers 2, 3, 5, and 6), and are defined by their functional circuit contributions, rather than on the basis of gross morphology. Such cell types may represent the closest homology with associational networks found in mammalian extrastriate cortex and their expression in the mesopallium suggests that MVL may be the visual nucleus in the tectofugal pathway at which category-level representation of objects emerges. In support of categorical representation in MVL, Marzluff et al. (2012) used PET brain imaging to demonstrate that crows exhibit significant neuronal activation of the anterior mesopallium in response to viewing human faces. Moreover, projections from ENTO to MVL in pigeons are arranged along an anterior–posterior axis (Krützfeldt and Wild, 2005), suggestive of a functional continuation of the parallel visual processing streams for form/colour (anterior) and motion (posterior) at the level of the nRT of the thalamus (Wang et al., 1993), ENTO (Nguyen et al., 2004) and into the telencephalon. Azizi et al. (2019) recently demonstrated that representations of animate (humans and animals) vs. inanimate objects (natural and artificial objects) could be extracted from population responses using a linear discriminant analysis at the level of MVL, but not from ENTO. Interestingly, the sub-category of human faces and body parts was identified to be driving the linear increase in classification performance for animate-category stimuli, indicating that MVL exhibits a population code of features for the representation of biologically relevant object categories. We performed similar multivariate styles of analysis to Azizi et al. (2019) to assess if visual forebrain structures would exhibit a population coding of conspecifics faces, but failed to demonstrate a significant population representation of pigeon faces as a category using RSM (see Figure 5), or any relative differences in firing rates elicited by pigeon faces and non-face stimuli between ENTO, MVL, NFL, or TPO.

The absence of any category-selective responses may also be explained by some critical differences in the functional organisation of the tectofugal visual pathway at the level of the association structures. For example, Stacho et al. (2016) determined that MVL, NFL, and TPO were activated equally during form/motion discrimination tasks, and showed no significant differences between hemisphere or anteroposterior position. These findings may reflect a neuronal organisation in birds where parallel processing streams for form/motion are segregated up to primary telencephalic centres (such as ENTO; Nguyen et al., 2004) which subsequently fuse and integrate both form/motion and other multimodal aspects of tectofugal visual information upstream in association forebrain areas to save processing space (Stacho et al., 2016). Our analysis of visual responsivity patterns showed that there were no significant differences in activation between ENTO, NFL, MVL, and TPO, or between hemispheres for each region. While posterior ENTO showed significantly greater visual responsivity than anterior ENTO (see Table 4), there was little evidence for anteroposterior differences in activation at the level of MVL. The combined evidence suggests that the association visual forebrain of birds may integrate multimodal sensory representations using mechanisms that differ substantially from mammals. Further electrophysiological and fMRI studies are required to determine what computations ENTO, MVL, NFL, and TPO may contribute to object representation, and further examine their proposed homology with extrastriate visual cortex.

## Conclusion

We are rapidly approaching a comprehensive understanding of object recognition in macaques, humans, and other mammalian species. How non-mammalian vertebrates solve the problems of object representation without a neocortex is a long-standing problem in evolutionary neuroscience, though multiple lines of accumulating evidence suggest that these abilities arise from circuitry fundamentally similar to extrastriate cortex. While we found no evidence of face-selective neurons in ENTO, NFL, MVL, or TPO, birds’ predisposition to attend to the local features of stimuli in visual discrimination tasks likely influenced our pigeons’ perception of objects as a global configuration of feature dimensions. It remains to be determined how the nuclear architecture of the tectofugal visual forebrain constructs stable representations of ethologically relevant percepts, and how the output of such circuits contribute to birds visual behaviour.

## Data Availability

The raw data supporting the conclusions of the study will be made available by the authors upon request, without undue reservation, to any qualified researcher.

## Author Contributions

WC designed the study and performed electrophysiological data collection, MATLAB data analysis, histology, and wrote the manuscript. BP lead MATLAB data analysis and gave invaluable manuscript guidance. MC assisted with the design of the study, wrote all behavioural task programmes to perform the study, read drafts, assisted with the structure of the manuscript, and provided the resources to conduct the study.

## Conflict of Interest Statement

The authors declare that the research was conducted in the absence of any commercial or financial relationships that could be construed as a potential conflict of interest. The handling Editor and reviewer GZ declared their involvement as co-editors in the Research Topic and confirm the absence of any other collaboration.

## References

[B1] Atoji Y. Karim M. R. (2014). Homology of the mesopallium in the adult chicken identified by gene expression of the neocortical marker cholecystokinin. *Neurosci. Lett.* 562 85–89. 10.1016/j.neulet.2014.01.011 24440787

[B2] Aust U. Braunöder E. (2015). Transfer between local and global processing levels by pigeons (*Columba livia*) and humans (Homo sapiens) in exemplar-and rule-based categorization tasks. *J. Compar. Psychol.* 129:1. 10.1037/a0037691 25150965

[B3] Azizi A. H. Pusch R. Koenen C. Klatt S. Bröcker F. Thiele S. (2019). Emerging category representation in the visual forebrain hierarchy of pigeons (*Columba livia*). *Behav. Brain Res.* 356 423–434. 10.1016/j.bbr.2018.05.014f 29885380

[B4] Bao P. Tsao D. Y. (2018). Representation of multiple objects in macaque category-selective areas. *Nat. Commun.* 9:1774. 10.1038/s41467-018-04126-7 29720645 PMC5932008

[B5] Behroozi M. Ströckens F. Stacho M. Güntürkün O. (2017). Functional connectivity pattern of the internal hippocampal network in awake pigeons: a resting-state fMRI study. *Brain. Behav. Evol.* 90 62–72. 10.1159/000475591 28866684

[B6] Bilkey D. K. Russell N. Colombo M. (2003). A lightweight microdrive for single-unit recording in freely moving rats and pigeons. *Methods* 30 152–158. 10.1016/S1046-2023(03)00076-8 12725781

[B7] Briscoe S. D. Albertin C. B. Rowell J. J. Ragsdale C. W. (2018). Neocortical association cell types in the forebrain of birds and alligators. *Curr. Biol.* 28 686–696. 10.1016/j.cub.2018.01.036 29456143 PMC11098552

[B8] Cavoto K. K. Cook R. G. (2001). Cognitive precedence for local information in hierarchical stimulus processing by pigeons. *J. Exp. Psychol. Anim. Behav. Process.* 27:3. 10.1037//0097-7403.27.1.3 11199512

[B9] Chang L. Tsao D. Y. (2017). The code for facial identity in the primate brain. *Cell* 169 1013–1028. 10.1016/j.cell.2017.05.011 28575666 PMC8088389

[B10] Cook R. G. Wright A. A. Drachman E. E. (2013). Categorization of birds, mammals, and chimeras by pigeons. *Behav. Process.* 93 98–110. 10.1016/j.beproc.2012.11.006 23174337 PMC3815572

[B11] De Groof G. Jonckers E. Güntürkün O. Denolf P. Van Auderkerke J. Van der Linden A. (2013). Functional MRI and functional connectivity of the visual system of awake pigeons. *Behav. Brain Res.* 239 43–50. 10.1016/j.bbr.2012.10.044 23137696

[B12] Domjan M. Greene P. North N. C. (1989). Contextual conditioning and the control of copulatory behavior by species-specific sign stimuli in male Japanese quail. *J. Exp. Psychol. Anim. Behav. Process.* 15:147. 10.1037/0097-7403.15.2.147 2708940

[B13] Domjan M. Nash S. (1988). Stimulus control of social behaviour in male Japanese quail, *Coturnix coturnix* japonica. *Anim. Behav.* 36 1006–1015. 10.1016/S0003-3472(88)80060-5

[B14] Fisher C. Freiwald W. A. (2015). Contrasting specializations for facial motion within the macaque face-processing system. *Curr. Biol.* 25 261–266. 10.1016/j.cub.2014.11.038 25578903 PMC4302012

[B15] Freiwald W. A. Tsao D. Y. (2010). Functional compartmentalization and viewpoint generalization within the macaque face-processing system. *Science* 330 845–851. 10.1126/science.1194908 21051642 PMC3181095

[B16] Freiwald W. A. Tsao D. Y. Livingstone M. S. (2009). A face feature space in the macaque temporal lobe. *Nat. Neurosci.* 12:1187. 10.1038/nn.2363 19668199 PMC2819705

[B17] Güntürkün O. Koenen C. Iovine F. Garland A. Pusch R. (2018). The neuroscience of perceptual categorization in pigeons: a mechanistic hypothesis. *Learn. Behav.* 46 229–241. 10.3758/s13420-018-0321-6 29532328

[B18] Gutiérrez-Ibáñez C. Iwaniuk A. N. Wylie D. R. (2018). Parrots have evolved a primate-like telencephalic-midbrain-cerebellar circuit. *Sci. Rep.* 8:9960. 10.1038/s41598-018-28301-4 29967361 PMC6028647

[B19] Hawkins J. Ahmad S. Cui Y. (2017). A theory of how columns in the neocortex enable learning the structure of the world. *Front. Neural Circuits* 11:81. 10.3389/fncir.2017.00081 29118696 PMC5661005

[B20] Hodos W. Karten H. J. (1970). Visual intensity and pattern discrimination deficits after lesions of ectostriatum in pigeons. *J. Compar. Neurol.* 140 53–68. 10.1002/cne.901400104 5459212

[B21] Hung C. C. Yen C. C. Ciuchta J. L. Papoti D. Bock N. A. Leopold D. A. (2015). Functional mapping of face-selective regions in the extrastriate visual cortex of the marmoset. *J. Neurosci.* 35 1160–1172. 10.1523/JNEUROSCI.2659-14.2015 25609630 PMC4300322

[B22] Husband S. A. Shimizu T. (1999). Efferent projections of the ectostriatum in the pigeon (*Columba livia*). *J. Compar. Neurol.* 406 329–345. 10.1002/(SICI)1096-9861(19990412)406:3<329::AID-CNE3>3.0.CO;2-A 10102499

[B23] Issa E. B. DiCarlo J. J. (2012). Precedence of the eye region in neural processing of faces. *J. Neurosci.* 32 16666–16682. 10.1523/JNEUROSCI.2391-12.2012 23175821 PMC3542390

[B24] Jarvis E. D. Güntürkün O. Bruce L. Csillag A. Karten H. Kuenzel W. (2005). Avian brains and a new understanding of vertebrate brain evolution. *Nat. Rev. Neurosci.* 6:151. 10.1038/nrn1606 15685220 PMC2507884

[B25] Johnston M. Anderson C. Colombo M. (2017). Pigeon NCL and NFL neuronal activity represents neural correlates of the sample. *Behav. Neurosci.* 131:213. 10.1016/j.bbr.2016.10.003 28471222

[B26] Karten H. J. Hodos W. (1967). *Stereotaxic Atlas of the Brain of the Pigeon (Columba livia)*. Baltimore, MD: THE Johns Hopkins Press.

[B27] Kendrick K. M. Baldwin B. A. (1987). Cells in temporal cortex of conscious sheep can respond preferentially to the sight of faces. *Science* 236 448–450. 10.1126/science.3563521 3563521

[B28] Keppel G. Zedeck S. (1982). *Design and Analysis: A Researcher’s Handbook.* Englewood Cliffs, NJ: Prentice-Hall, Inc.

[B29] Kiani R. Esteky H. Mirpour K. Tanaka K. (2007). Object category structure in response patterns of neuronal population in monkey inferior temporal cortex. *J. Neurophysiol.* 97 4296–4309. 10.1152/jn.00024.2007 17428910

[B30] Kimberly R. P. Holden A. L. Bamborough P. (1971). Response characteristics of pigeon forebrain cells to visual stimulation. *Vision Res.* 11:475 10.1016/0042-6989(71)90088-5 5558930

[B31] Koenen C. Pusch R. Bröker F. Thiele S. Güntürkün O. (2016). Categories in the pigeon brain: a reverse engineering approach. *J. Exp. Anal. Behav.* 105 111–122. 10.1002/jeab.179 26615363

[B32] Kornblith S. Cheng X. Ohayon S. Tsao D. Y. (2013). A network for scene processing in the macaque temporal lobe. *Neuron* 79 766–781. 10.1016/j.neuron.2013.06.015 23891401 PMC8127731

[B33] Kriegeskorte N. Mur M. Bandettini P. A. (2008a). Representational similarity analysis-connecting the branches of systems neuroscience. *Front. Syst. Neurosci.* 2:4. 10.3389/neuro.06.004.2008 19104670 PMC2605405

[B34] Kriegeskorte N. Mur M. Ruff D. A. Kiani R. Bodurka J. Esteky H. (2008b). Matching categorical object representations in inferior temporal cortex of man and monkey. *Neuron* 60 1126–1141. 10.1016/j.neuron.2008.10.043 19109916 PMC3143574

[B35] Krützfeldt N. O. Wild J. M. (2005). Definition and novel connections of the entopallium in the pigeon (*Columba livia*). *J. Compar. Neurol.* 490 40–56. 10.1002/cne.20627 16041718

[B36] Marzluff J. M. Miyaoka R. Minoshima S. Cross D. J. (2012). Brain imaging reveals neuronal circuitry underlying the crow’s perception of human faces. *Proc. Natl. Acad. Sci. U.S.A.* 109 15912–15917. 10.1073/pnas.1206109109 22984177 PMC3465369

[B37] Mayer U. Rosa-Salva O. Lorenzi E. Vallortigara G. (2016). Social predisposition dependent neuronal activity in the intermediate medial mesopallium of domestic chicks (*Gallus gallus* domesticus). *Behav. Brain Res.* 310 93–102. 10.1016/j.bbr.2016.05.019 27173429

[B38] Moeller S. Crapse T. Chang L. Tsao D. Y. (2017). The effect of face patch microstimulation on perception of faces and objects. *Nat. Neurosci.* 20:743. 10.1038/nn.4527 28288127 PMC8086516

[B39] Moeller S. Freiwald W. A. Tsao D. Y. (2008). Patches with links: a unified system for processing faces in the macaque temporal lobe. *Science* 320 1355–1359. 10.1126/science.1157436 18535247 PMC8344042

[B40] Nakamura T. Croft D. B. Westbrook R. F. (2003). Domestic pigeons (*Columba livia*) discriminate between photographs of individual pigeons. *Anim. Learn. Behav.* 31 307–317. 10.3758/BF03195993 14733480

[B41] Nasr S. Tootell R. B. (2012). Role of fusiform and anterior temporal cortical areas in facial recognition. *Neuroimage* 63 1743–1753. 10.1016/j.neuroimage.2012.08.031 23034518 PMC3472036

[B42] Nguyen A. P. Spetch M. L. Crowder N. A. Winship I. R. Hurd P. L. Wylie D. R. (2004). A dissociation of motion and spatial-pattern vision in the avian telencephalon: implications for the evolution of “visual streams”. *J. Neurosci.* 24 4962–4970. 10.1523/JNEUROSCI.0146-04.2004 15163688 PMC6729365

[B43] Patton T. B. Szafranski G. Shimizu T. (2010). Male pigeons react differentially to altered facial features of female pigeons. *Behaviour* 147 757–773. 10.1163/000579510X491090

[B44] Peissig J. J. Young M. E. Wasserman E. A. Biederman I. (2019). Pigeons spontaneously form three-dimensional shape categories. *Behav. Process.* 158 70–76. 10.1016/j.beproc.2018.11.003 30439476 PMC10631373

[B45] Pinsk M. A. Arcaro M. Weiner K. S. Kalkus J. F. Inati S. J. Gross C. G. (2009). Neural representations of faces and body parts in macaque and human cortex: a comparative FMRI study. *J. Neurophysiol.* 101 2581–2600. 10.1152/jn.91198.2008 19225169 PMC2681436

[B46] Quiroga R. Q. (2016). Neuronal codes for visual perception and memory. *Neuropsychologia* 83 227–241. 10.1016/j.neuropsychologia.2015.12.016 26707718

[B47] Reiner A. Perkel D. J. Bruce L. L. Butler A. B. Csillag A. Kuenzel W. (2004). Revised nomenclature for avian telencephalon and some related brainstem nuclei. *J. Compar. Neurol.* 473 377–414. 10.1002/cne.20118 15116397 PMC2518311

[B48] Scarf D. Stuart M. Johnston M. Colombo M. (2016). Visual response properties of neurons in four areas of the avian pallium. *J. Compar. Physiol. A* 202 235–245. 10.1007/s00359-016-1071-6 26868923

[B49] Schalk G. Kapeller C. Guger C. Ogawa H. Hiroshima S. Lafer-Sousa R. (2017). Facephenes and rainbows: causal evidence for functional and anatomical specificity of face and color processing in the human brain. *Proc. Natl. Acad. Sci. U.S.A.* 114 12285–12290. 10.1073/pnas.1713447114 29087337 PMC5699078

[B50] Schultz W. (2016). Dopamine reward prediction-error signalling: a two-component response. *Nat. Rev. Neurosci.* 17:183. 10.1038/nrn.2015.26 26865020 PMC5549862

[B51] Shanahan M. Bingman V. P. Shimizu T. Wild M. Güntürkün O. (2013). Large-scale network organization in the avian forebrain: a connectivity matrix and theoretical analysis. *Front. Comput. Neurosci.* 7:89. 10.3389/fncom.2013.00089 23847525 PMC3701877

[B52] Shimizu T. Bowers A. N. (1999). Visual circuits of the avian telencephalon: evolutionary implications. *Behav. Brain Res.* 98 183–191. 10.1016/S0166-4328(98)00083-7 10683106

[B53] Soto F. A. Wasserman E. A. (2011). Asymmetrical interactions in the perception of face identity and emotional expression are not unique to the primate visual system. *J. Vision* 11 24–24. 10.1167/11.3.24 21454855

[B54] Soto F. A. Wasserman E. A. (2012). Visual object categorization in birds and primates: integrating behavioral, neurobiological, and computational evidence within a “general process” framework. *Cognit. Affect. Behav. Neurosci.* 12 220–240. 10.3758/s13415-011-0070-x 22086545

[B55] Stacho M. Ströckens F. Xiao Q. Güntürkün O. (2016). Functional organization of telencephalic visual association fields in pigeons. *Behav. Brain Res.* 303 93–102. 10.1016/j.bbr.2016.01.045 26802723

[B56] Tsao D. Y. Freiwald W. A. Knutsen T. A. Mandeville J. B. Tootell R. B. (2003). Faces and objects in macaque cerebral cortex. *Nat. Neurosci.* 6:989. 10.1038/nn1111 12925854 PMC8117179

[B57] Tsao D. Y. Freiwald W. A. Tootell R. B. Livingstone M. S. (2006). A cortical region consisting entirely of face-selective cells. *Science* 311 670–674. 10.1126/science.1119983 16456083 PMC2678572

[B58] Tyrrell L. P. Butler S. R. Yorzinski J. L. Fernández-Juricic E. (2014). A novel system for bi-ocular eye-tracking in vertebrates with laterally placed eyes. *Methods Ecol. Evol.* 5 1070–1077. 10.1111/2041-210X.12249

[B59] Ungerleider L. G. Haxby J. V. (1994). ‘What’ and ‘where’ in the human brain. *Curr. Opin. Neurobiol.* 4 157–165. 10.1016/0959-4388(94)90066-3 8038571

[B60] Vallortigara G. (1992). Right hemisphere advantage for social recognition in the chick. *Neuropsychologia* 30 761–768. 10.1016/0028-3932(92)90080-6 1407491

[B61] Vallortigara G. Andrew R. J. (1994). Differential involvement of right and left hemisphere in individual recognition in the domestic chick. *Behav. Process.* 33 41–57. 10.1016/0376-6357(94)90059-0 24925239

[B62] Verhaal J. Kirsch J. A. Vlachos I. Manns M. Güntürkün O. (2012). Lateralized reward-related visual discrimination in the avian entopallium. *Eur. J. Neurosci.* 35 1337–1343. 10.1111/j.1460-9568.2012.08049.x 22452655

[B63] Wang Y. C. Jiang S. Frost B. J. (1993). Visual processing in pigeon nucleus rotundus: luminance, color, motion, and looming subdivisions. *Vis. Neurosci.* 10 21–30. 10.1017/S0952523800003199 8424926

[B64] Watanabe S. Ito Y. (1990). Discrimination of individuals in pigeons. *Bird Behav.* 9 20–29. 10.3727/015613890791749136

[B65] Xiao Q. Güntürkün O. (2009). Natural split-brain?: Lateralized memory for task contingencies in pigeons. *Neurosci. Lett.* 458 75–78. 10.1016/j.neulet.2009.04.030 19379793

[B66] Xiao Q. Li D. P. Wang S. R. (2006). Looming-sensitive responses and receptive field organization of telencephalic neurons in the pigeon. *Brain Res. Bull.* 68 322–328. 10.1016/j.brainresbull.2005.09.003 16377438

[B67] Yamazaki Y. Aust U. Huber L. Hausmann M. Güntürkün O. (2007). Lateralized cognition: asymmetrical and complementary strategies of pigeons during discrimination of the “human concept”. *Cognition* 104 315–344. 10.1111/j.1460-9568.2012.08049.x 16905127

[B68] Yamins D. L. Hong H. Cadieu C. F. Solomon E. A. Seibert D. DiCarlo J. J. (2014). Performance-optimized hierarchical models predict neural responses in higher visual cortex. *Proc. Natl. Acad. Sci. U.S.A.* 111 8619–8624. 10.1073/pnas.1403112111 24812127 PMC4060707

[B69] Yue X. Pourladian I. S. Tootell R. B. Ungerleider L. G. (2014). Curvature-processing network in macaque visual cortex. *Proc. Natl. Acad. Sci. U.S.A.* 111 E3467–E3475. 10.1073/pnas.1412616111 25092328 PMC4143055

